# Association between insomnia and the incidence of myocardial infarction: A systematic review and meta‐analysis

**DOI:** 10.1002/clc.23984

**Published:** 2023-02-25

**Authors:** Yomna E. Dean, Mohamed A. Shebl, Samah S. Rouzan, Bdoor Ahmed A Bamousa, Nesreen Elsayed Talat, Sana Afreen Ansari, Yousef Tanas, Muaaz Aslam, Sara Gebril, Taher Sbitli, Ramy Eweis, Rameen Shahid, Amr Salem, Heba Ahmed Abdelaziz, Jaffer Shah, Walaa Hasan, Diaa Hakim, Hani Aiash

**Affiliations:** ^1^ Faculty of Medicine Alexandria University Alexandria Egypt; ^2^ Faculty of Medicine Cairo University, Kasr Al‐ Ainy Cairo Egypt; ^3^ College of Medicine Alfaisal University Riyadh Saudi Arabia; ^4^ Deccan College of Medical Sciences Hyderabad India; ^5^ Shaikh Khalifa Bin Zayed Al‐Nahyan Medical and Dental College Lahore Pakistan; ^6^ Faculty of Medicine Ain Shams University Cairo Egypt; ^7^ Faculty of Medicine Beni Suef University Beni Suef Egypt; ^8^ Dow International Medical College Karachi Pakistan; ^9^ Department of Family Health, High Institute of Public Health Alexandria University Alexandria Egypt; ^10^ Department of Public Health New York State Department of Health New York United States; ^11^ Faculty of Medicine Suez Canal University Ismailia Egypt; ^12^ Department of Cardiology, Brigham and Women's Hospital Harvard Medical School Boston United States; ^13^ Department of Surgery, Cardiovascular Perfusion, and Medicine SUNY Upstate Medical University Syracuse United States

**Keywords:** insomnia, myocardial infarction, sleep disorders, STEMI

## Abstract

**Background:**

Insomnia has been closely associated with cardiovascular disease (CVD) including myocardial infarction (MI). Our study aims to assess the eligibility of insomnia as a potential risk factor for MI.

**Methods:**

PubMed, Scopus, and Web of Science were searched using terms; such as “Insomnia” and “MI.” Only observational controlled studies with data on the incidence of MI among insomniacs were included. Revman software version 5.4 was used for the analysis.

**Results:**

Our pooled analysis showed a significant association between insomnia and the incidence of MI compared with noninsomniacs (relative risk [RR] = 1.69, 95% confidence interval [CI] = 1.41–2.02, *p* < .00001). Per sleep duration, we detected the highest association between ≤5 h of sleep, and MI incidence compared to 7−8 h of sleep (RR = 1.56, 95% CI = 1.41–1.73). Disorders of initiating and maintaining sleep were associated with increased MI incidence (RR = 1.13, 95% CI = 1.04–1.23, *p* = .003). However, subgroup analysis of nonrestorative sleep and daytime dysfunction showed an insignificant association with MI among both groups (RR = 1.06, 95% CI = 0.91–1.23, *p* = .46). Analysis of age, follow‐up duration, sex, and comorbidities showed a significant association in insomniacs.

**Conclusion:**

Insomnia and ≤5 h of sleep are highly associated with increased incidence of MI; an association comparable to that of other MI risk factors and as such, it should be considered as a risk factor for MI and to be incorporated into MI prevention guidelines.

## Graphical abstract

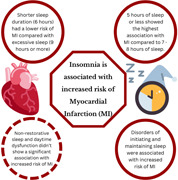

## 1 INTRODUCTION

Insomnia is the most common sleep disorder and is known to negatively impact the general health of the population and the quality of life.^1^, ^2^ Due to its difficulty to treat, it has contributed to a significant socioeconomic burden on a global scale; as many of its sufferers have reported decreased productivity and absenteeism at work.^3^ In the modern era, insomnia is becoming increasingly common with a prevalence of 10%–15% in the United States.^2^

There have been multiple studies linking insomnia to the increased risk of cardiovascular and metabolic diseases.^4^, ^5^ Among insomniacs, there is an alteration of the hypothalamic‐pituitary‐adrenal axis with higher levels of adrenocorticotropic hormone (ACTH) and cortisol compared to healthy individuals.^6^ Elevation of cortisol has been associated with myocardial infarction (MI); whereas individuals who suffered from an acute MI, have been shown to have higher levels of cortisol in the month preceding their MI compared with healthy individuals.^7^ An experiment conducted on mice has shown that chronic stress and elevated cortisol led to the acceleration of atherosclerosis which could potentially lead to MI.^8^ Insomniacs suffer from both chronic stress and elevated cortisol due to the lack of sleep, which exacerbates their risk of developing MI.

As of the time of writing this paper, Insomnia isn't considered a risk factor for MI^9^; our study aims to analyze the current literature and assess the eligibility of insomnia as a risk factor for MI.

## 2 METHODS

The protocol for this paper was registered on PROSPERO (CRD42022348707) and the regulations of the preferred reporting items of systematic reviews and meta‐analyses (PRISMA) were followed.^10^

### 2.1 Search strategy

A literature search of the following databases (PubMed, Scopus, and Web of Science) on May 17, 2022, using key terms such as “Insomnia,” “sleep complaints,” “sleep initiation,” “Myocardial Infarction,” “MI,” and “STEMI,” was performed to identify studies of interest. (View the supplementary material for the full search strategy).

### 2.2 Insomnia definition

Insomnia was defined according to the International Classification of Diseases, Ninth Revision, Clinical Modification (ICD‐9‐CM); codes 307.4 (a disorder of initiating or maintaining sleep) and 780.5 (insomnia, unspecified). We also utilized the Diagnostic and Statistical Manual of Mental Disorders (DSM‐5), which stated that insomnia is a sleep disorder characterized by the presence of any of the following three symptoms: (1) difficulty initiating sleep, (2) difficulty maintaining sleep, (3) early morning awakening with inability to return to sleep.^11^ In addition to that, we included an objective measure of insomnia which is the sleep duration.^12^ Obstructive sleep apnea (OSA) and other sleep apneas weren't included in our study.

### 2.3 Inclusion and exclusion criteria

We screened the studies according to the following criteria:

Inclusion criteria: Studies selected must be in the English language; controlled observational studies with data on the incidence of myocardial infarction among adults (≥18 years) suffering from insomnia, including cross‐sectional, case–control, and cohort studies were included.

Exclusion criteria: Editorials, commentaries, reviews, systematic reviews, meta‐analyses, case reports, case series, animal studies, studies without data about the risk of myocardial infarction in patients suffering from insomnia, and studies lacking a control group were excluded; in case of duplicate studies, the most recent study with the largest study population was included.

### 2.4 Study selection

Two independent reviewers (T.S. and M.A.) screened the studies according to our criteria. If a consensus is not achieved, a third independent reviewer (R.S.) was consulted to resolve the conflict.

### 2.5 Data extraction and quality assessment

Each study was extracted by two reviewers independently (R.E. and S.G.). The data was then compared to confirm accuracy. If a consensus is not achieved, a third independent reviewer (R.S.) was consulted to resolve the conflict.

For the baseline and summary, the following data were extracted from the eligible studies: the last name of the first author, year of publication, study design, number of participants, age of participants, sex of participants, average body mass index, the prevalence of smoking, the prevalence of alcohol, and baseline diseases.

For the outcomes, the following data were extracted: MI incidence, sleep duration, DIMS, nonrestorative sleep and daytime dysfunction, age, sex, hypertension, diabetes, and hyperlipidemia among insomnia and noninsomnia groups.

The risk of bias was assessed utilizing Newcastle‐Ottawa Scale (NOS) items,^13^ with a total score of nine points, to evaluate the quality of observational studies. We defined the observational studies with a NOS score of ≥7 stars as high quality and NOS score of <7 stars as low quality.

## 3 RESULTS

### 3.1 Literature search

**Figure 1:**
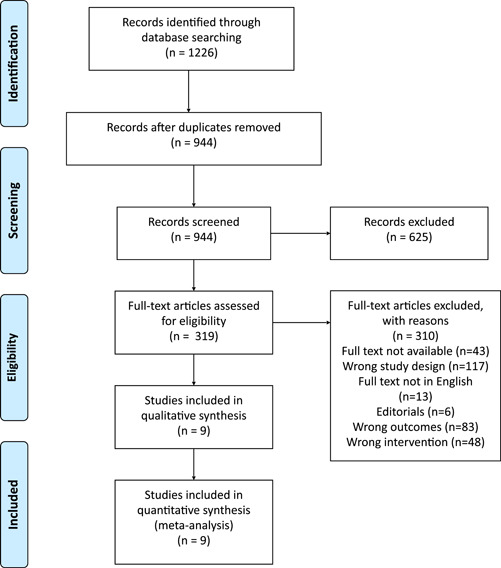
PRISMA flow diagram showing the study selection process. Characteristics of the included studies.

A complete search of the literature yielded 1226 studies, and after duplicate removal, there were 944 studies eligible for the title and abstract screening. Of the 944, 625 were irrelevant and 319 studies were eligible for full‐text screening. Finally, 9^14^, ^16^, ^17^, ^18^, ^19^, ^20^, ^21^, ^22^ studies were included in the meta‐analysis after the full‐text screening, as shown in the PRISMA diagram (Figure 1).

The total number of patients included in the study is 1 184 256 patients; 153 881 patients in the insomnia group, and 1 030 375 patients in the noninsomnia group. A summary of the included studies and patients' baseline data are shown in Table 1. The overall quality of the included studies was high as shown in (Supporting Information: Table S1).

**Table 1:** Baseline characteristics and summary of the included studies.

Author (Year)	Country	Study design	Follow‐up duration (years)	Sample size	Age, mean (SD)	Male, no. (%)	Smoking, No. (%)	BMI, mean (SD)	Alcohol No. (%)	Baseline diseases	Conclusion
Chung 2013	Taiwan	Prospective	9–13	147 297	51.3 (16.7)	53 547	‐	‐	‐	DM	This nationwide population‐based cohort study provides evidence that patients with non‐apnea sleep disorders are at higher risk of
										HTN	
										Hyperlipidemia	developing acute coronary syndrome.
		Cohort								COPD	
										CVA	
Elwood 2006	UK	Prospective Cohort	10	1874	‐	1874 (100)	637	26.8 (3.7)	‐	‐	The risk of an ischemic stroke is increased in men whose sleep is frequently disturbed, and
							(34)				
											daytime sleepiness is associated with a significant increase in ischemic heart disease events.
Hsu 2015	Taiwan	Prospective Cohort	10	44 080	47.7 (15.7)	18 912 (42.9)	‐	‐	‐	Hyperlipidemia	Insomnia is associated with an increased risk of future cardiovascular events.
										HTN	
										Depression	
										CPD	
										PAD	
										CHF	
										CKD	
										CAD	
										DM	
										Hyperlipidemia	
Laugsand	Norway	Prospective Cohort	11.4	51 982	49.4 (16.8)	23 226 (44.6)	14 611 (28.1)	26.3 (4.0)	29 278 (56.3)	DM	Insomnia is associated with a moderately increased risk for AMI.
										HTN Dyslipidemia	
										Depression	
										Anxiety	
Meisinger 2007	Germany	Prospective Cohort	10.1	6896	57.4 (8.0)	3508 (50.8)	1250 (18.1)	27.7 (4.1)	4692 (68.0)	Dyslipidemia	Modest associations between short sleep duration and
										HTN Depression	
											difficulties maintaining sleep and incident MI were seen in middle‐aged
										DM	
											women but not men from the general population.
Schwartz 1998	US	Prospective Cohort	3	2960	73	993 (33.5)	518 (17.5)	‐	‐	DM	A subjective sleep complaint increases the likelihood of a first MI in older adults
										HTN	
											without overt coronary heart disease independently of classic coronary risk factors and appears
										Obesity	
											to be a marker for a syndrome of depression and malaise that may have a causal relationship to MI.
Zheng 2019	China	Prospective Cohort	9.6	487 200	51.0	199 241 (40.9)	130 335 (26.7)	23.6	73 634 (15.1)	HTN	Individual and coexisting insomnia symptoms are independent risk factors for CVD incidence,
										DM	
										Anxiety Depression	especially among young adults or adults who have not developed hypertension.
Daghlas 2019	UK	Prospective Cohort	7.04	461 347	‐	‐	‐	‐	‐	Hypothyroidism Hyperthyroidism	Prospective observational and MR analyses support short sleep duration as a potentially causal risk factor for MI. Investigation of sleep extension to prevent MI may be warranted.
										Migraine	
										Rheumatoid Arthritis	
										Osteoarthritis	
										Deep vein thrombosis	
										COPD	
Kalmbach 2016	US	Cross‐sectional	‐	3911	46.0 (13.3)	1357 (34.7)	‐	28.1	‐	DM	Insomnia disorder with short sleep is the most severe phenotype of insomnia and comorbid with many cardiometabolic and psychiatric
										HTN	
										Hyperlipidemia	
								(6.4)			
											illnesses, whereas morbidity profiles are highly similar between insomniacs with normal sleep duration and former insomniacs.


Abbreviations: AMI, acute myocardial infarction; BMI, body mass index; CAD, coronary artery disease; CHF, congestive heart failure; CKD, chronic kidney disease; COPD, chronic obstructive pulmonary disease; CPD, chronic pulmonary disease; CVA, cerebrovascular disease; DM, diabetes mellitus; HTN, hypertension; MI, myocardial infarction; MR, Mendelian randomization; No., number; PAD, peripheral artery disease; SD, standard deviation.

### 3.2 Outcomes

#### 3.2.1 MI incidence (insomnia vs. noninsomnia)

**Figure 2:**
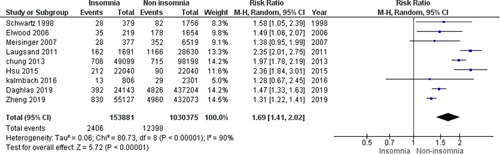
Forest plot of the comparison between insomnia and the incidence of MI. MI, myocardial infarction.

Our pooled analysis revealed a statistically significant association between insomnia and an increased incidence of MI. Patients with insomnia were at 1.69 times greater risk for the development of MI (relative risk [RR] = 1.69, 95% confidence interval [CI] = 1.41–2.02, *p* < .00001). We detected a significant heterogeneity among studies (*p* < .00001, *I* ^2^ = 90%) that was not solved by the leave‐one‐out test, Figure 2.

#### 3.2.2 MI incidence and sleep duration

**a. Five hours or less versus 6 h**

**Figure 3:**
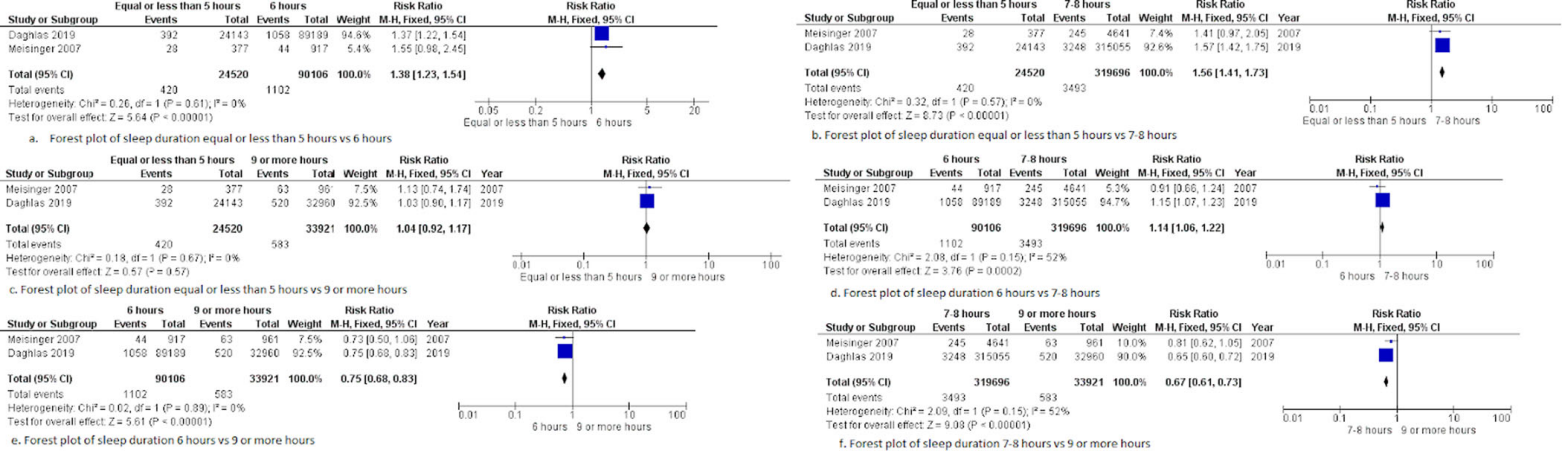
Forest plot of the comparison between sleep duration and the incidence of MI. MI, myocardial infarction.

Our pooled analysis revealed a statistically significant association between sleep duration and increased incidence of MI. Patients who slept 5 h or less were at 1.38 times greater risk for MI (RR = 1.38, 95% CI = 1.23–1.54, *p* < .00001) compared to patients who slept 6 h. We observed no heterogeneity among studies (*p* = .61, *I* ^2^ = 0%), Figure 3A.

**b. 5 hours or less versus 7–8 h**

Our pooled analysis revealed a statistically significant association between sleep duration and increased incidence of MI. Patients who slept 5 h or less were at 1.56 times greater risk for MI (RR = 1.56, 95% CI = 1.41–1.73, *p* < .00001) compared to patients who slept 7–8 h. We observed no heterogeneity among studies (*p* = .57, *I* ^2^ = 0%), Figure 3B.

**c. 5 hours or less versus 9 h or more**

Our pooled analysis showed no statistically significant difference between 5 h or less and 9 h or more of sleep regarding the incidence of MI (RR = 1.04, 95% CI = 0.92–1.17, *p* = .57). We observed no heterogeneity among studies (*p* = .67, *I* ^2^ = 0%), Figure 3C.

**d. 6 hours versus 7–8 h**

Our pooled analysis revealed a statistically significant association between sleep duration and increased incidence of MI. Patients who slept 6 h were at 1.14 times greater risk for MI (RR = 1.14, 95% CI = 1.06–1.22, *p* = .0002) compared to patients who slept 7–8 h. We observed no heterogeneity among studies (*p* = .15, *I* ^2^ = 52%), Figure 3D.

**e. 6 hours versus 9 h or more**

Our pooled analysis revealed a statistically significant association between 9 h or more of sleep duration and an increased incidence of MI. Patients who slept 6 h were at decreased risk of myocardial infarction compared to patients who slept 9 or more hours (RR = 0.75, 95% CI = 0.68–0.83, *p* < .00001). We observed no heterogeneity among studies (*p* = .89, *I* ^2^ = 0%), Figure 3E.

**f. 7–8 hours versus 9 h or more**

Our pooled analysis revealed a statistically significant association between 9 h or more of sleep and an increased incidence of MI. Patients who slept 7–8 h were at decreased risk of myocardial infarction compared to patients who slept 9 or more hours (RR = 0.67, 95% CI = 0.61–0.73, *p* < .00001). We observed no heterogeneity among studies (*p* = .15, *I* ^2^ = 52%), Figure 3F.

#### 3.2.3 Subgroup analysis of the follow‐up duration (≤5 years vs. >5 years)

**Figure 4:**
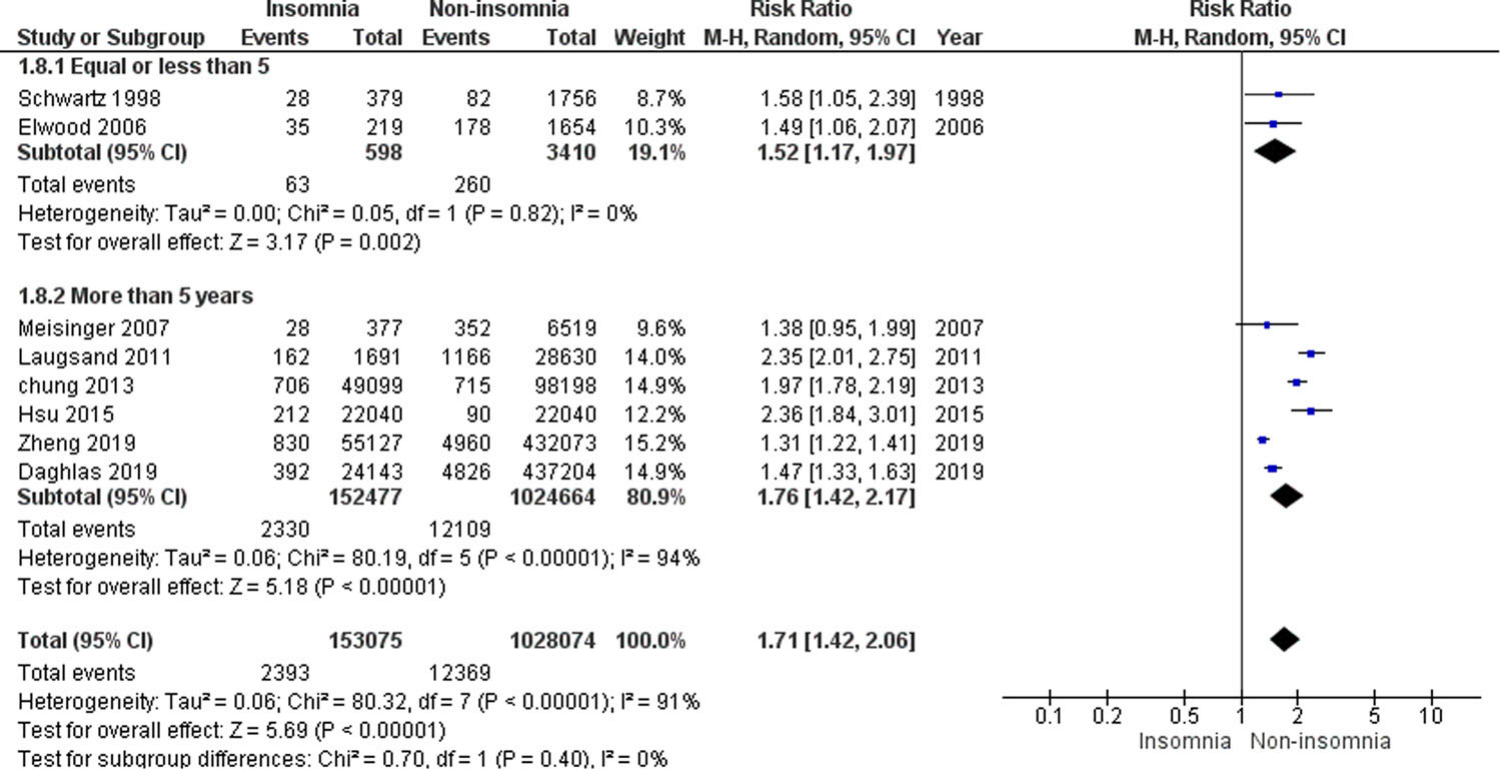
Forest plot of insomnia and the incidence of MI comparison between ≤5 years versus >5 years follow‐up duration subgroups. MI, myocardial infarction.

In the subgroup of “follow‐up ≤5 years,” the pooled analysis revealed a statistically significant association between insomnia and increased incidence of MI (RR = 1.52, 95% CI = 1.17–1.97, *p* = .002), with no heterogeneity among studies (*p* = .82, *I* ^2^ = 0%), Figure 4.

In the subgroup of “follow‐up >5 years,” the pooled analysis revealed a statistically significant association between insomnia and increased incidence of MI (RR = 1.76, 95% CI = 1.42–2.17, *p* < .00001), with significant heterogeneity among studies (*p* < .00001, *I* ^2^ = 94%) that was not solved by the leave‐one‐out test, Figure 4.

### 3.3 DIMS subgroup

Our pooled analysis showed a statistically significant association between DIMS and increased incidence of MI. Patients with DIMS had 1.13 times greater risk for MI compared to the control group (RR = 1.13, 95% CI = 1.04–1.23, *p* = .003), with no observed heterogeneity among studies (*p* = .58, *I* ^2^ = 0%) (Supporting Information: Figure S1).

#### 3.3.1 Nonrestorative sleep and daytime dysfunction subgroup

Our pooled analysis showed no statistically significant difference between the nonrestorative sleep and daytime dysfunction group and control group regarding the incidence of MI (RR = 1.06, 95% CI = 0.91–1.23, *p* = .46). We observed no heterogeneity among studies (*p* = .57, *I* ^2^ = 0%) (Supporting Information: Figure S1).

#### 3.3.2 Age (<65 vs. ≥65 years subgroups)

In the <65 years age subgroup, our pooled analysis revealed a statistically significant association between insomnia and increased incidence of MI compared with the control group (RR = 1.68, 95% CI = 1.40–2.02, *p* < .00001), with significant heterogeneity among studies (*p* < .00001, *I* ^2^ = 89%) that was not solved by the leave‐one‐out test (Supporting Information: Figure S2).

In the ≥65 years age subgroup, our pooled analysis revealed a statistically significant association between insomnia and increased incidence of MI (RR = 2.06, 95% CI = 1.52–2.79, *p* < .00001), with significant heterogeneity among studies (*p* = .12, *I* ^2^ = 60%) (Supporting Information: Figure S2).

### 3.4 Sex subgroups

In the male subgroup, our pooled analysis revealed a statistically significant association between insomnia and increased incidence of MI (RR = 2.03, 95% CI = 1.78–2.33, *p* < .00001), with no significant heterogeneity among studies (*p* = .82, *I* ^2^ = 0%) (Supporting Information: Figure S3).

In the female subgroup, our pooled analysis revealed a statistically significant association between insomnia and increased incidence of MI (RR = 2.24, 95% CI = 1.54–3.25, *p* < .0001), with no significant heterogeneity among studies (*p* = .08, *I* ^2^ = 68%) (Supporting Information: Figure S3).

### 3.5 Comorbidities subgroups

In the hypertension subgroup, the pooled analysis revealed a statistically significant association between insomnia and increased incidence of MI (RR = 1.84, 95% CI = 1.16–2.90, *p* = .009), with significant heterogeneity among studies (*p* = .006, *I* ^2^ = 87%) (Supporting Information: Figure S4).

In the diabetes subgroup, our pooled analysis revealed a statistically significant association between insomnia and increased incidence of MI (RR = 2.06, 95% CI = 1.05–4.04, *p* = .04), with significant heterogeneity among studies (*p* = .006, *I* ^2^ = 87%) (Supporting Information: Figure S4).

In the hyperlipidemia subgroup, our pooled analysis revealed a statistically significant association between insomnia and increased incidence of MI (RR = 1.76, 95% CI = 1.04–2.99, *p* = .04), we detected a significant heterogeneity among studies (*p* = .03, *I* ^2^ = 79%) (Supporting Information: Figure S4).

## 4 DISCUSSION

Our study demonstrates that insomniacs are at a higher risk of developing MI relative to noninsomniacs. According to the DSM‐5 criteria of insomnia, DIMS was significantly associated with a higher risk of MI, while nonrestorative sleep and daytime dysfunction yielded an insignificant association. Per sleep duration, patients who slept 5 h or less had the highest association with MI incidence, compared to those who slept 7−8 h. Longer sleep duration (9 h or more) wasn't protective against MI; quite to the contrary, patients who slept 6 h were at a lower risk of MI, compared to those who slept 9 h or more. Subgroup analysis of the follow‐up duration revealed that both short (5 years or less) and long (more than 5 years) durations were associated with a significant increase in the risk of MI. Both male and female insomniacs were at a higher risk of MI compared with noninsomniacs. Lastly, the presence of insomnia in addition to a comorbid disease (hypertension, dyslipidemia, or diabetes) was associated with a higher risk of MI.

Concerning the current literature and its accordance with our results, Sofi et al.^23^ in their meta‐analysis, have shown that insomnia is associated with an increased risk of cardiovascular morbidity and mortality (RR = 1.45, 95% CI = 1.29−1.62). It is important to note that their search was up to 2011, and it only included 122 501 individuals; since then, multiple controlled observational studies have been published regarding this matter. After the inclusion of these studies, we managed to reach a sample size of 1, 184, 256 and analyze individual insomnia symptoms and various sleep durations.

Our results support Hu et al.^24^ findings which concluded that DIMS is associated with a higher risk of CVD including myocardial infarction. On the other hand, they reported that non‐restorative sleep is significantly associated with a higher risk of CVD. This finding contradicts our subgroup analysis which showed that nonrestorative sleep and daytime dysfunction are associated with an insignificant increase in the risk of MI. Another study conducted by He et al.^25^ reported a similar finding that nonrestorative sleep is associated with a higher risk of cardio‐cerebral vascular events.

Regarding sleep duration, Chandola et al.^26^ stated that a short sleep duration of 5 h, wasn't significantly associated with an increased risk of coronary artery disease (CAD) including MI. This opposes our results, as we reported that sleeping 5 h or less is highly associated with an increased risk of MI compared to those who slept 7−8 h. Our findings are reinforced by Ayas et al.^27^ and Nagi et al.^28^ who concluded that a sleep duration of 5 h or less is associated with a higher risk of developing CAD. Our analysis has shown that longer sleep durations of 9 h or more could lead to an increased incidence of MI; a similar conclusion was demonstrated by Jike et al.,^29^ in their meta‐analysis, which showed that long sleep hours are associated with a higher risk of CAD (RR = 1.24, 95% CI = 1.13–1.37).

Miguel‐Yanes et al.^30^ and Alkhouli et al.^31^ reported that MI incidence was higher among men. Our study reported that among insomniacs, there is an increased risk of MI in both men and women. Notwithstanding, the risk of MI in our study was higher among female insomniacs.

Our study showed that regardless of age, insomniacs are associated with a higher risk of MI. Lian et al.^32^ cross‐sectional study reinforce our findings; they concluded that short sleep duration is associated with a higher risk of MI in patients <65 and >65 years old. Older age is usually associated with a higher risk of MI^33^; this is supported by the findings in our study.

### 4.1 Strengths

Our study has a cohort of 1 184 256 individuals, originating from six different countries (United States, United Kingdom, Norway, Germany, Taiwan, and China) and three different continents, increasing the generalizability of our findings. All of our nine included studies are controlled; eight of them are prospective cohorts; this allows us to establish causality and consider insomnia as a risk factor for MI. Additionally, we performed a subgroup analysis according to the confounding factors (age, sex, and comorbidities); enabling us to reach a stronger conclusion. Another subgroup analysis based on individual insomnia symptoms and sleep duration was conducted, to reach a more individualized conclusion that can be applied to patients suffering from different symptoms of insomnia.

### 4.2 Limitations

There was significant heterogeneity among the included studies; this could be attributed to the different sample sizes, ages, and follow‐up duration.

In most studies, data were obtained from questionnaires, therefore, biases may have occurred due to misinterpretation of the questions proposed.

Finally, research papers that were published in any language other than English (e.g., Chinese) were excluded and thus were not a part of the Systematic Review and Meta‐analysis.

## 5 CONCLUSION AND POLICY IMPLICATIONS

Insomnia has been significantly associated with an increased incidence of myocardial infarction. Subsequently, insomnia should be integrated into guidelines on the primary prevention of cardiovascular disease.

## CONFLICT OF INTEREST STATEMENT

The authors declare no conflict of interest.

## Supporting information

Supporting information.

Supporting information.

Supporting information.

## Data Availability

Data available upon request from the corresponding author.
