# Supplementary material for: Association between insomnia and the incidence of myocardial infarction: A systematic review and meta‐analysis
Source: Clin Cardiol. 2023 Feb 25;46(4):376–85. doi: 10.1002/clc.23984 (PMC10106668; doi:10.1002/clc.23984)
Supplement: Supplementary file 1 — Supporting information. [file CLC-46-376-s001.docx]

**PubMed (17th May)**

(insomnia[Title/Abstract] OR "sleep complaints"[Title/Abstract] OR "sleep initiation"[Title/ Abstract] OR "sleep disorders"[Title/Abstract] OR "sleep disturbances"[Title/Abstract] OR "disorders of initiating and maintaining sleep"[Title/Abstract] OR "poor sleep quality"[Title/ Abstract]) AND ("Myocardial infarction"[Title/Abstract] OR "MI"[Title/Abstract] OR "NSTEMI"[Title/Abstract] OR "STEMI"[Title/Abstract] OR "acute coronary syndrome"[Title/ Abstract])

**Scopus (17th May)**

( TITLE-ABS-KEY ( insomnia OR "sleep complaints" OR "sleep initiation" OR "sleep disturbances" OR "disorders of initiating and maintaining sleep" OR "poor sleep quality" ) AND TITLE-ABS-KEY ( "Myocardial infarction" ) )

**Web of science (17th May)**

(insomnia Or "sleep complaints" or "sleep initiation" or "sleep disorders" or "sleep disturbances" or "poor sleep quality" or "Sleep Initiation and Maintenance Disorders") AND ("Myocardial infarction" or "coronary artery disease")
