# Supplementary material for: Association between insomnia and the incidence of myocardial infarction: A systematic review and meta‐analysis
Source: Clin Cardiol. 2023 Feb 25;46(4):376–85. doi: 10.1002/clc.23984 (PMC10106668; doi:10.1002/clc.23984)

Figure S1: Forest plot of Difficulty Initiating and Maintaining Sleep (DIMS), Non-restorative sleep and Daytime Dysfunction and incidence of myocardial infarction


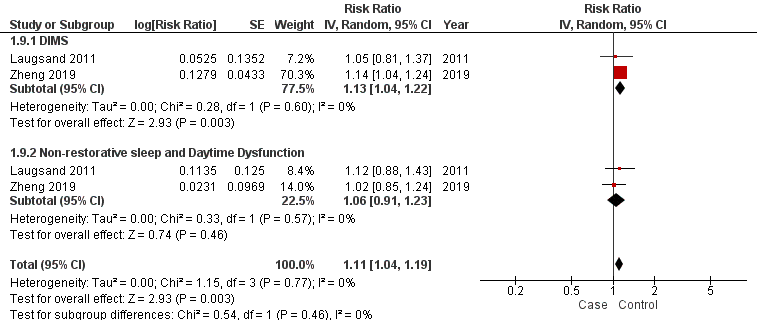


Figure S2: Forest plot of the comparison between less than 65 years subgroup and more than 65 years subgroup in age outcome in incidence of myocardial infarction


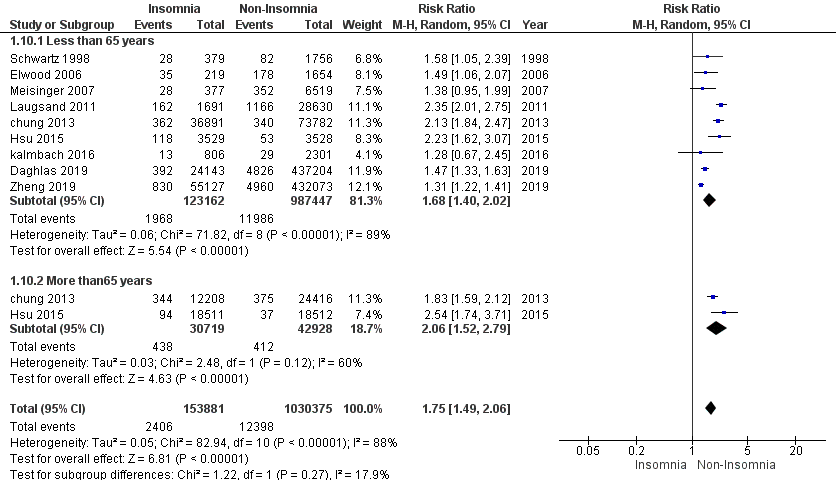


Figure S3: Forest plot of the comparison between male and female subgroups in the association between insomnia and the incidence of myocardial infarction.


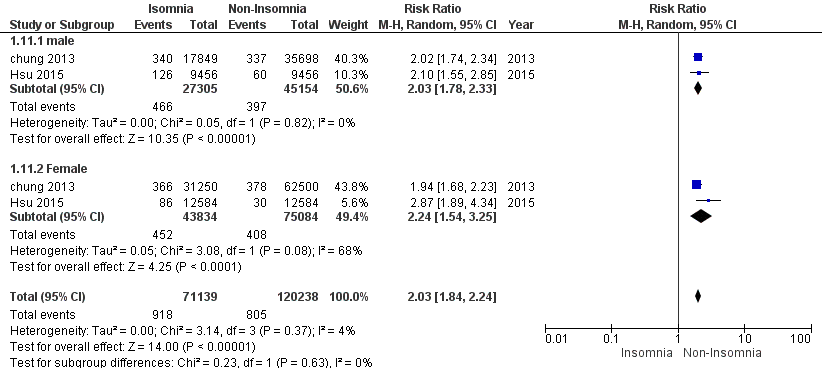


Figure S4: Forest plot of the comparison between subgroups hypertension, diabetes and hyperlipidemia subgroups in comorbidities outcome in incidence of myocardial infarction


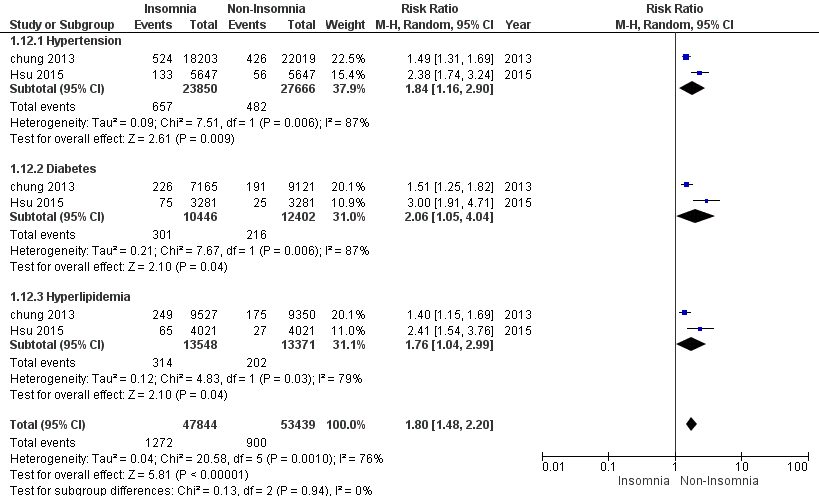

Supplement: Supplementary file 2 — Supporting information. [file CLC-46-376-s003.docx]
