# Supplementary material for: Association between insomnia and the incidence of myocardial infarction: A systematic review and meta‐analysis
Source: Clin Cardiol. 2023 Feb 25;46(4):376–85. doi: 10.1002/clc.23984 (PMC10106668; doi:10.1002/clc.23984)
Supplement: Supplementary file 3 — Supporting information. [file CLC-46-376-s002.docx]

Table 1: Quality Assessment of the included studies

| Author (Year) | Representativeness | Selection of the non-exposed cohort | Ascertainment of exposure | Demonstration that the outcome of interest was not present at start of study | Comparability | Assessment of the outcome | Was follow-up long enough for outcomes to occur? | Adequacy of follow-up of cohorts | Total points | Score |
| --- | --- | --- | --- | --- | --- | --- | --- | --- | --- | --- |
| Chung 2013 | 1 | 1 | 1 | 1 | 2 | 1 | 1 | 1 | 9 | High |
| Elwood 2006 | 0 | 1 | 0 | 1 | 2 | 1 | 1 | 1 | 7 | High |
| Hsu 2015 | 1 | 1 | 1 | 1 | 2 | 1 | 1 | 1 | 9 | High |
| Laugsand 2011 | 1 | 1 | 0 | 1 | 2 | 1 | 1 | 1 | 8 | High |
| Meissinger 2007 | 1 | 1 | 1 | 1 | 2 | 1 | 1 | 1 | 9 | High |
| Schwartz 1998 | 1 | 1 | 1 | 1 | 2 | 0 | 0 | 1 | 7 | High |
| Zheng 2019 | 1 | 1 | 1 | 1 | 2 | 1 | 1 | 1 | 9 | High |
| Daghlas 2019 | 1 | 1 | 0 | 1 | 2 | 1 | 1 | - | 7 | High |
| Kalmbach 2016 | 0 | 1 | 1 | 0 | 2 | 0 | 0 | 0 | 4 | Low |
